# IGF2BP3 promotes the progression of colorectal cancer and mediates cetuximab resistance by stabilizing EGFR mRNA in an m^6^A-dependent manner

**DOI:** 10.1038/s41419-023-06099-y

**Published:** 2023-09-01

**Authors:** Li-Jie Chen, Hui-Ye Liu, Zhi-Yuan Xiao, Ting Qiu, Dan Zhang, Ling-Jie Zhang, Fang-Yi Han, Guo-Jun Chen, Xue-Mei Xu, Jiong-Hua Zhu, Yan-Qing Ding, Shu-Yang Wang, Ya-Ping Ye, Hong-Li Jiao

**Affiliations:** 1 Department of Pathology, Nanfang Hospital, Southern Medical University, Guangzhou, China; 2 Department of Pathology, School of Basic Medical Sciences, Southern Medical University, Guangzhou, China; 3 Guangdong Provincial Key Laboratory of Molecular Tumor Pathology, Guangzhou, China; 4 Department of Pathology, Shenzhen People’s Hospital, Second Clinical Medical College of Jinan University, Shenzhen, Guangdong China

**Keywords:** Colorectal cancer, Cancer epidemiology, Mechanisms of disease, Oncogenes

## Abstract

Insulin-like growth factor 2 mRNA-binding protein 3 (IGF2BP3), an RNA-binding protein, is associated with tumorigenesis and progression. However, the exact molecular mechanisms of IGF2BP3 in colorectal cancer (CRC) oncogenesis, progression, and drug resistance remain unclear. This study found that IGF2BP3 was upregulated in CRC tissues. Clinically, the elevated IGF2BP3 level is predictive of a poor prognosis. Functionally, IGF2BP3 enhances CRC tumorigenesis and progression both in vitro and in vivo. Mechanistically, IGF2BP3 promotes epidermal growth factor receptor (EGFR) mRNA stability and translation and further activates the EGFR pathway by serving as a reader in an N6-methyladenosine (m^6^A)-dependent manner by cooperating with METTL14. Furthermore, IGF2BP3 increases the drug resistance of CRC cells to the EGFR-targeted antibody cetuximab. Taken together, our results demonstrated that IGF2BP3 was a functional and clinical oncogene of CRC. Targeting IGF2BP3 and m^6^A modification may therefore offer rational therapeutic targets for patients with CRC.

## Introduction

Colorectal cancer (CRC) was estimated to be the third most commonly diagnosed cancer and the second leading cause of cancer-related deaths worldwide in 2020 [1]. Despite the improved substantial diagnostic and therapeutic strategies, the incidence of CRC remains high. Moreover, CRC gradually presents the characteristics of younger onset age [2], a higher degree of malignancy, and more drug resistance. Thus, understanding the molecular mechanisms of CRC initiation and progression is essential for early diagnoses and effective therapy.

Insulin-like growth factor 2 mRNA-binding proteins (IGF2BPs), including IGF2BP1/2/3, are RNA-binding proteins (RBPs) that serve as posttranscriptional regulatory factors for mRNA stability and translation [3]. Over the past few years, studies have increasingly documented the contribution of IGF2BPs in fundamental processes in cancer biology. Among these, IGF2BP3 has been implicated in various aspects of human tumor progression regulating cell growth, migration, and response to drugs [4]. IGF2BP3 is upregulated and potentially oncogenic in various tumor types, such as bladder cancer [5], lung cancer [6], gastric cancer [7], and so forth. Furthermore, IGF2BP3 has been identified as a promising biomarker for various cancers including colon cancer [8, 9]. However, the molecular mechanism of IGF2BP3 in CRC remains unclear.

Epidermal growth factor receptor (EGFR), also known as ERBB1, belongs to the ERBB family of cell-surface receptor tyrosine kinases. Abnormal, enhanced expression of EGFR triggers a series of intracellular signals, ultimately leading to the proliferation of cancer cells, induction of angiogenesis, and metastasis [10]. Although the EGFR-targeted antibodies cetuximab and panitumumab have provided substantial benefits to patients with advanced cancer, their clinical efficacy is limited by intrinsic and acquired resistance [11]. Therefore, understanding the molecular bases of resistance to EGFR blockade in CRC is crucial.

As the most abundant mRNA modification, N6-methyladenosine (m^6^A) modification participates in almost all steps of RNA metabolism, including mRNA translation, degradation, splicing, export, and folding, resulting in the alteration of target gene expression [12]. M^6^A plays critical roles in multiple fundamental biological processes such as cell differentiation, tissue development, and tumorigenesis [3]. The abundance and effects of m^6^A on RNA are determined by the dynamic interplay between its methyltransferases (writers), binding proteins (readers), and demethylases (erasers) [13]. In this regard, readers refer to proteins that can recognize and bind to m^6^A sites, leading to different destinies of target RNA, including translation, mRNA stability, nuclear trafficking, microRNA binding, and RNA–protein interaction [14].

In the present study, we identified the oncogenic role of IGF2BP3. We found that IGF2BP3 was highly expressed in CRC, and the upregulation of IGF2BP3 was associated with a poor prognosis of patients with CRC. The overexpression of IGF2BP3 enhanced CRC tumorigenesis and progression in vitro and in vivo. We demonstrated EGFR as a target gene of IGF2BP3 and that IGF2BP3 regulated EGFR expression by serving as a reader to stabilize m6A-modified EGFR mRNA. In addition, we provided evidence that IGF2BP3 was associated with drug resistance to the EGFR-targeted antibody cetuximab in CRC.

## Results

### IGF2BP3 is upregulated in CRC

**Fig. 1:**
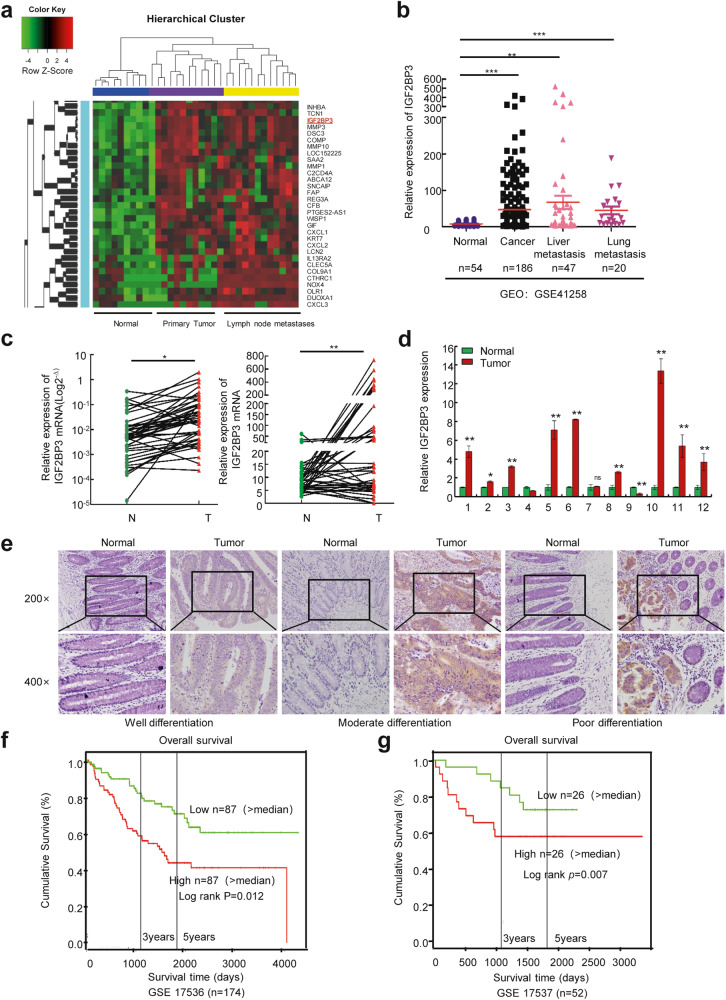
IGF2BP3 is up-regulated in colorectal cancer. **a** Different expressed genes in 11 pairs of colorectal cancer patients with normal intestinal mucosa, primary tumor, and lymph node metastasis tissues. **b** Expression of IGF2BP3 in normal tissue, colorectal cancer, liver metastasis, and lung metastasis from GSE41258 dataset. **c** Left: relative expression of IGF2BP3 mRNA in 47 cases of fresh colorectal cancer paired normal intestinal tissues. Right: relative expression of IGF2BP3 mRNA in colorectal cancer tissues and matched normal tissues in TCGA Database. **d** Quantitation of relative protein expression of IGF2BP3 in 12 cases of fresh CRC tissues by Western blot. The expression is normalized by α-tubulin. **e** Representative IHC images of IGF2BP3 in well, moderate, and poor-differentiated CRC tissues. **f**, **g** Kaplan–Meier survival curves of CRC patients with high or low IGF2BP3 expression from GSE17536 (**f**) and GSE17537 (**g**) dataset. *p < 0.05; **p < 0.01; ***p < 0.001.

We conducted RNA sequencing (RNA-seq) to compare the gene expression profiles of 11 pairs of primary tumors and their corresponding normal intestinal mucosa and lymph node metastases from patients with CRC. We identified that IGF2BP3, an RNA-binding protein, was significantly upregulated in primary tumors and lymph node metastasis (Fig. 1a). Further analysis of data from Gene Expression Omnibus data base (GEO) (GSE41258 and GSE71187), Oncomine (www.oncomine.com), and the cBioPortal for Cancer Genomics databases suggested that the expression of IGF2BP3 was higher in CRC than in normal tissues (Fig. 1b and S1a–c). Quantitative Real-time Polymerase Chain Reaction (qRT-PCR) and Western blot further confirmed the upregulation of IGF2BP3 in CRC (Fig. 1c, d and S1d). The immunohistochemistry (IHC) staining results also proved that the high expression of IGF2BP3 was significantly correlated with poorer differentiation (Fig. 1e).

In 1983, Pierce shared the idea that carcinogenesis was an epigenetic event, similar to postembryonic differentiation [15]. Previous studies revealed that IGF2BP3 was expressed in most organs during embryogenesis, but either became absent or was expressed at extremely low levels in most tissues after birth [16]. Therefore, we analyzed the expression profile chip of mouse early colon embryonic development (GSE38831). Previous findings showed a gradual decrease in IGF2BP3 expression with embryonic development (Fig. S1e). In addition, the analysis of GSE71187 showed that the expression of IGF2BP3 was higher in both embryonic and CRC tissues than in normal intestinal mucosa tissues (Fig. S1b). IGF2BP3 showed a spatiotemporal expression pattern of “high expression during embryogenesis, inhibited expression in the normal intestinal mucosa, and upregulated expression in CRC tissues,” suggesting that IGF2BP3 might be involved in tumorigenesis and tumor progression of CRC.

Moreover, the interrogation of the GEO dataset (GSE17536 and GSE17537) revealed that a higher level of IGF2BP3 was significantly associated with shorter overall survival in patients with CRC (Fig. 1f, g). The elevated expression of IGF2BP3 was also significantly associated with certain clinicopathological features such as Tumor Node Metastasis (TNM) staging and Dukes staging (Table S1). Taken together, these results suggested that IGF2BP3 might serve as a prognostic biomarker for CRC.

### IGF2BP3 regulates the stability of EGFR mRNA

**Fig. 2:**
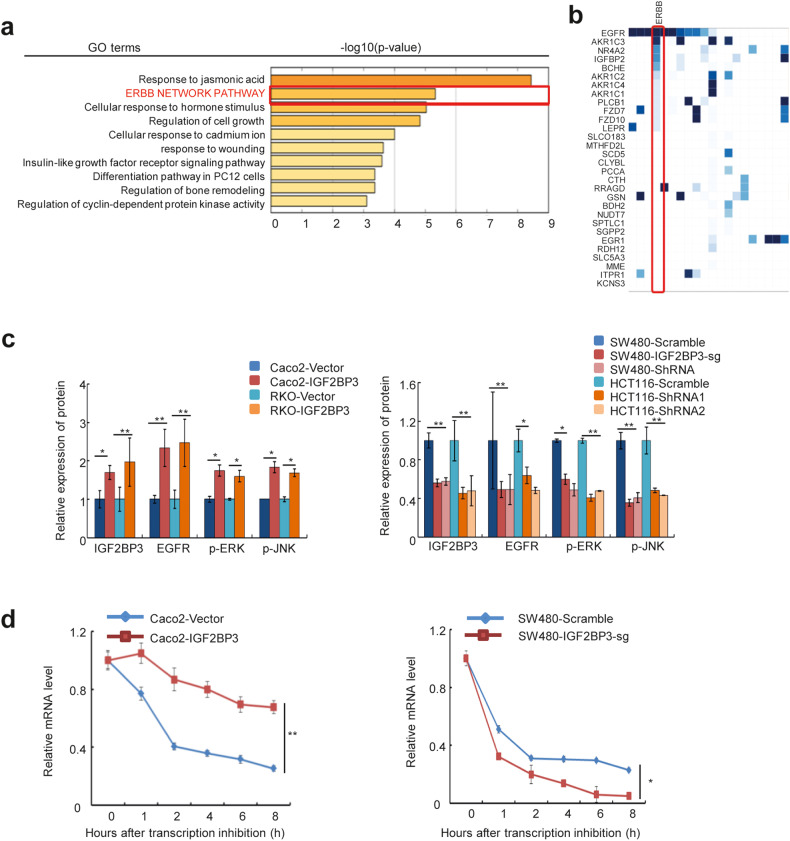
IGF2BP3 regulates the stability of EGFR mRNA. **a** Gene ontology and KEGG analysis of IGF2BP3 knockout SW480 cells. **b** Heat maps of key genes in each pathway. **c** The relative protein expressions of IGF2BP3, EGFR, p-ERK, p-JNK were measured by Western Blot in Caco2, RKO, SW480, and HCT116 cells after overexpression or knockdown/knockout of IGF2BP3, *n* = 3, nonparametric Mann Whitney test. **d** The EGFR mRNA half-life (t_1/2_) was detected by real-time PCR in Caco2 cells transfected with vector or IGF2BP3 (up) and SW480 cells transfected with scramble or IGF2BP3-sg (down). *n* = 3, nonparametric Mann–Whitney test. *p < 0.05; **p < 0.01; ***p < 0.001.

As an RNA-binding protein, IGF2BP3 promotes stability and translation by binding to the noncoding region of the target mRNA. To investigate the functional implications of IGF2BP3 and identify its downstream targets in CRC, we generated stable IGF2BP3-overexpressing and IGF2BP3-knockdown or knockout CRC cell lines (Fig. S2a–c). Gene Ontology (GO), Kyoto Encylopaedia of Genes and Genomes (KEGG) and Gene Set Enrichment Analysis (GSEA) of IGF2BP3 knockout SW480 cells revealed that the downregulated genes were significantly enriched in the ERBB (human epidermal growth factor receptor) Network Pathway gene set (Fig. 2a, b and Fig S2d). As one of the ERBB receptors, EGFR has been identified as an oncogene and was the first growth factor receptor to be proposed as a target for cancer therapy [10]. The analysis of the downstream genes of the EGFR pathway revealed that the overexpression of IGF2BP3 significantly upregulated the phosphorylated levels of p-ERK (phosphorylated extracellular regulated protein kinases, p-ERK) and p-JNK (phosphorylated c-Jun N-terminal kinase, p-JNK) (Fig. 2c and S2e). The data from the cBioPortal for Cancer Genomics databases suggested that the activation of EGFR was mostly induced by mutations, not by amplification (Fig. S2f). Then, we used the GSE41258 chip to analyze the expression of EGFR, which showed no significant difference in the expression of EGFR mRNA in normal and tumor tissues (Fig. S2g).

IGF2BP3 serves as a posttranscriptional regulatory factor for mRNA [17]. Therefore, we hypothesized that the regulation of EGFR protein by IGF2BP3 might be due to a difference in mRNA stability. The RNA stability curves showed that the knockdown or knockout of IGF2BP3 reduced the half-life of EGFR mRNA, while the overexpression of IGF2BP3 lengthened it (Fig. 2d).

Taken together, IGF2BP3 activated the EGFR signaling pathway by maintaining EGFR mRNA stability.

### IGF2BP3 stabilizes EGFR mRNA in an m^6^A-dependent manner

**Fig. 3:**
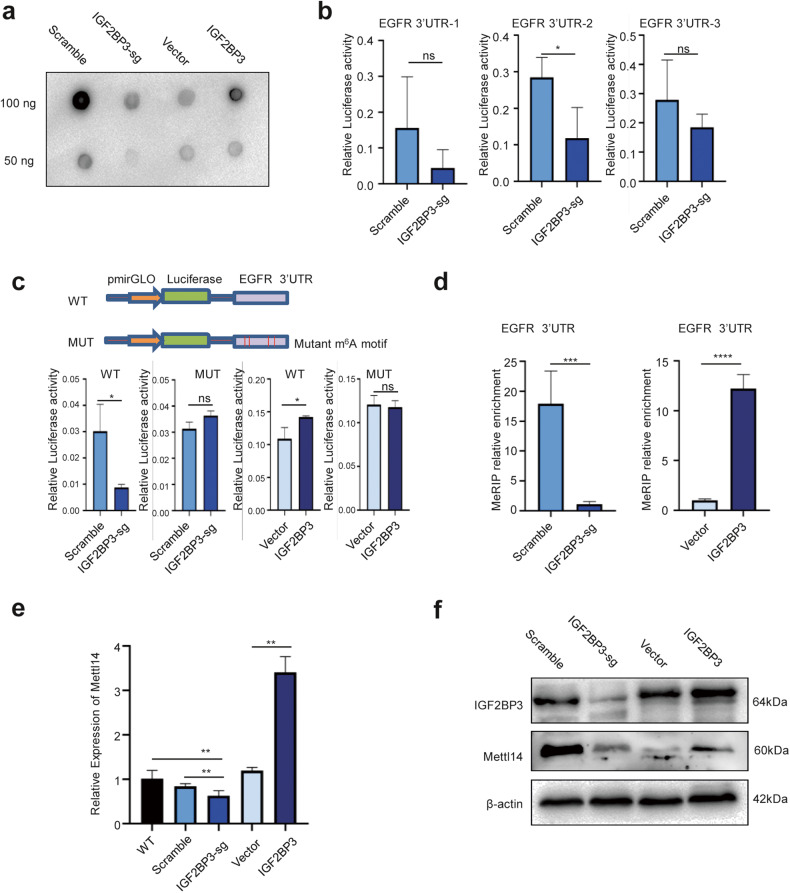
IGF2BP3 stabilizes EGFR mRNA in an m6A-dependent manner. **a** The m^6^A dot blot assay of global m^6^A abundance in mRNA of SW480 IGF2BP3-overexpressed and IGF2BP3-knockout cells. **b**, **c** Luciferase activity was measured in SW480 IGF2BP3-knockout cells and SW480 IGF2BP3-control cells transfected with the dual-luciferase reporter plasmids specifically targeting the three predicted m^6^A binding sites in the 3’UTR region of EGFR mRNA (**b**) and the luciferase reporters expressing WT or mutant human EGFR 3’UTRs (**c**). **d** MeRIP-qPCR analysis of EGFR 3’UTR m^6^A levels in SW480 IGF2BP3-overexpressed and IGF2BP3-knockout cells, *n* = 3, nonparametric Mann–Whitney test. **e**, **f** qRT-PCR (**e**) and Western blot assays (**f**) were performed to analyze the relative Mettl14 levels in SW480 IGF2BP3-overexpressed and IGF2BP3-knockout cells. sg, IGF2BP3-knockout. ns, no significance; *p < 0.05; **p < 0.01; ***p < 0.001; ****p < 0.0001.

Besides traditional RNA-binding sites (RBSs), a previous study demonstrated that IGF2BPs, acting as m^6^A readers, have the ability to bind to m^6^A RNAs and regulate their stability [3]. Consistently, m^6^A dot-blot analysis showed an evident relevance between IGF2BP3 and m^6^A levels (Fig. 3a). According to the previous finding that IGF2BP3 preferentially bound to the most common m^6^A motif ‘GGAC’ [3], we identified GGAC as the m^6^A consensus motif and showed that the GGAC motif was enriched in the 3′- Untranslated Regions (UTRs) of EGFR mRNA (Fig. S3a–c). We constructed both wild-type and mutant (m^6^A was replaced by T) EGFR luciferase reporter plasmids to further address the effect of m^6^A modification on EGFR mRNA (Fig. 3b, c). As expected, the knockout of IGF2BP3 substantially reduced the luciferase activity of the reporter constructs bearing the wild-type EGFR 3’-UTR with the second intact m^6^A site (Fig. 3b), whereas the overexpression of IGF2BP3 increased it (Fig. 3c). However, neither knockout nor overexpression of IGF2BP3 had a significant effect on luciferase activity reporter plasmids with m^6^A site mutations in SW480 cells (Fig. 3c). This finding was further supported by the methylated RNA Immunoprecipitation (MeRIP) assay, which showed that the knockout or overexpression of IGF2BP3 downregulated or upregulated, respectively, the m^6^A levels of EGFR in SW480 cells (Fig. 3d). Electrophoretic mobility shift analysis (EMSA) also validated the binding between IGF2BP3 and EGFR mRNA (Fig. S3d).

The functions of m^6^A in RNA metabolism are carried out by its readers, whereas the dynamic transcriptomic m^6^A modification is orchestrated by its writers and erasers [13]. Figure S3a shows the proportion of readers, writers, and erasers of m^6^A regulators [18]. We performed qPCR and Western blot assays to identify the corresponding m^6^A writer of the EGFR mRNA. The level of METTL14, an m^6^A writer, decreased in IGF2BP3-knockout cells and increased in IGF2BP3-overexpressing cells (Fig. 3e, f), indicating that METTL14 might be an m^6^A writer of EGFR mRNA.

Together, these results suggested that IGF2BP3 served as a reader and increased EGFR expression by stabilizing EGFR mRNA via an m^6^A-dependent mechanism partnered by METTL14.

### IGF2BP3 induces tumor cell proliferation and tumorigenesis depending on the expression of EGFR in CRC

**Fig. 4:**
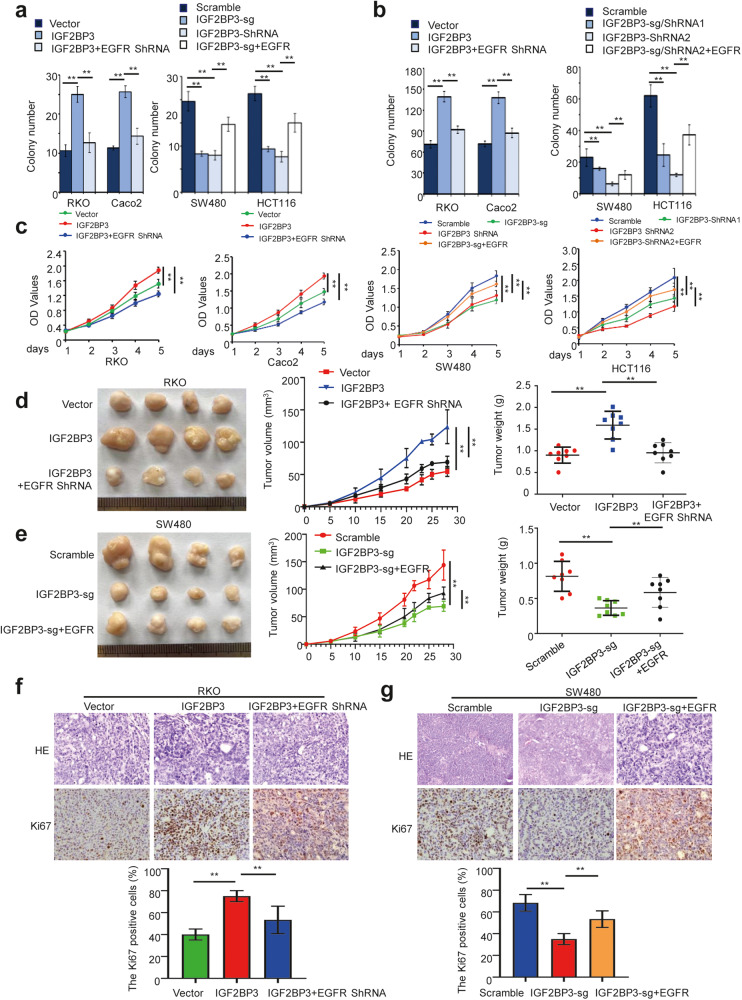
IGF2BP3 induces tumor cell proliferation and tumorigenesis depending on the expression of EGFR in CRC. **a**–**c** The ability of proliferation of CRC cells were evaluated by the colony formation assay (**a**), soft agar assay (**b**), and MTT assay (**c**) in the indicated cells with different treatments. **d**, **e** Representative gross images (left), tumor growth rate (middle), and tumor volume analysis (left) of subcutaneous tumors from the indicated groups. *n* = 8. **f**, **g** Representative HE staining and IHC images of Ki67 (up), and the percentage of Ki67 positive cells was quantitatively analyzed (down) in the indicated groups. Error bars represent the mean ± SD of 3 independent experiments; **p < 0.01.

Next, we verified the effect of IGF2BP3 on CRC cell proliferation and tumorigenesis. The overexpression of IGF2BP3 promoted cell proliferation and enhanced colony formation, whereas the knockdown/knockout of IGF2BP3 significantly suppressed cell proliferation and inhibited colony formation (Fig. 4a–c and S4a–d). Furthermore, the overexpression of IGF2BP3 dramatically increased tumor volumes in xenograft mouse models, whereas the knockout of IGF2BP3 effectively suppressed tumor growth (Fig. 4d–g).

In addition, we investigated whether EGFR participated in the biological function of IGF2BP3 in CRC. The downregulation of EGFR dramatically impaired IGF2BP3-induced cell proliferation, colony formation, and tumorigenesis, whereas the overexpression of EGFR partially restored the proliferation, colony formation, and tumorigenesis ability of IGF2BP3-knockdown cells (Fig. 4a–g and S4a–d), which supported EGFR as a critical target gene of IGF2BP3 in CRC.

Taken together, our results suggested that IGF2BP3 might promote tumor cell proliferation and tumorigenesis via activation of the EGFR signaling pathway.

### IGF2BP3 affects the sensitivity of CRC cells to cetuximab

**Fig. 5:**
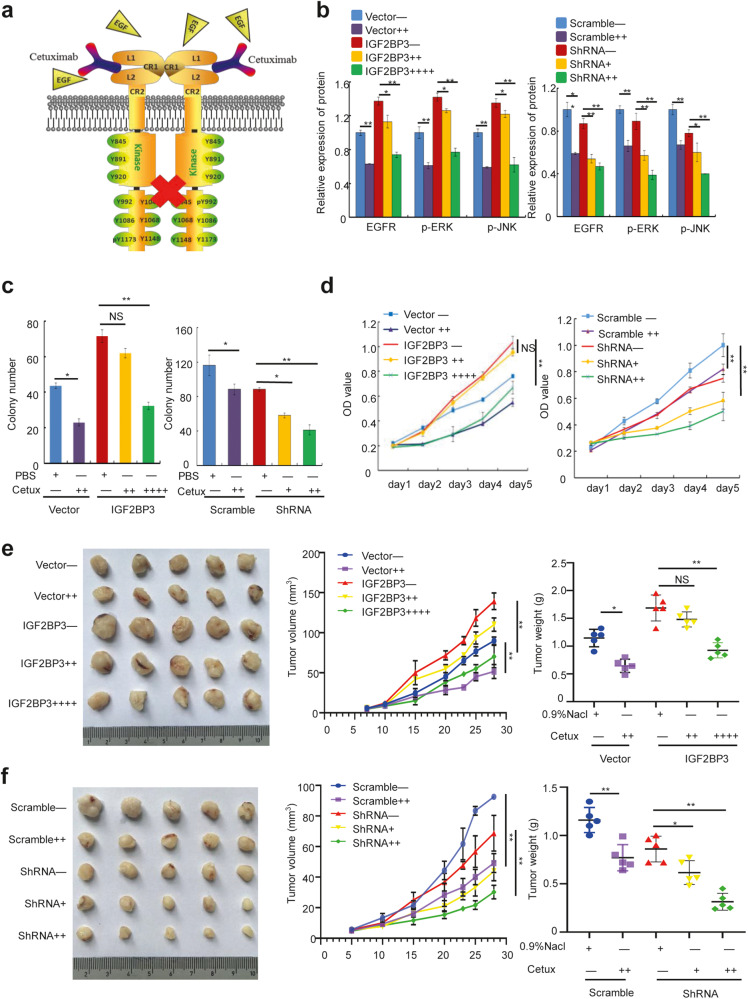
IGF2BP3 affects the sensitivity of colorectal cancer cells to cetuximab. **a** Cetuximab mechanism of blocking the activation of EGFR. **b** Western blot analysis and quantitation of the expression of EGFR and its downstream effector molecules in indicated groups of Caco2 cells. **c**, **d** Colony formation assay (**c**) and MTT assay (**d**) of Caco2 cells validated the effect of different cetuximab treatments on the proliferation rate in indicated groups. **e**, **f** Representative gross images (left), tumor growth rate (middle), and tumor volume analysis (left) in mice bearing Caco2 cells in different groups and treated with different drug concentrations. *n* = 5. “+” represents cetuximab at a concentration of 5 μg/μl, “++” represents cetuximab at a concentration of 10 μg/μl, “++++” represents cetuximab at a concentration of 20 μg/μl. Error bars represent the mean ± SD of 3 independent experiments; ns, no significance; *p < 0.05; **p < 0.01.

The EGFR-directed monoclonal antibodies cetuximab are now widely used in combination with chemotherapy or as a monotherapy for CRC (Fig. 5a). However, the resistance to cetuximab inevitably occurs, thereby limiting the clinical benefits of this drug. The analysis of the GSE56386 microarray from the GEO database and PRJEB34338 from the European Nucleotide Archive revealed that the expression of IGF2BP3 was significantly higher in patients who did not respond to cetuximab than in those who responded (Fig. S5a, b). The result was further validated by IHC in 30 clinical CRC samples without KRAS (Kirsten rat sarcoma viral oncogene homolog, KRAS) mutations (Fig. S5c). Therefore, we hypothesized that the high expression of IGF2BP3 might reduce drug response and induce drug resistance of CRC cells to cetuximab. The Western blot analysis revealed that adding cetuximab to cells in the control group significantly reduced the protein levels of EGFR and its downstream effector molecules. However, this effect of the same dose was less evident in IGF2BP3-overexpressing cells. Conversely, only a lower dose of cetuximab was required to cause significant downregulation of the expression of EGFR and its downstream target genes after interference with IGF2BP3 compared with the control group (Figs. S5d and 5b). In addition, we observed that cetuximab significantly suppressed cell proliferation and colony formation abilities, whereas the effect of the same dose was impaired in IGF2BP3-overexpressing cells and enhanced in IGF2BP3-knockdown cells (Fig. 5c, d and S5e, f). The results were further verified in xenograft mouse models, in which the ability of cetuximab to reduce tumor growth rate and volume was hindered by the overexpression of IGF2BP3 and promoted by the downregulation of IGF2BP3 (Fig. 5e, f). These data indicated that high IGF2BP3 expression increased drug resistance of CRC cells to cetuximab.

### IGF2BP3 level is clinically relevant in patients with CRC

**Fig. 6:**
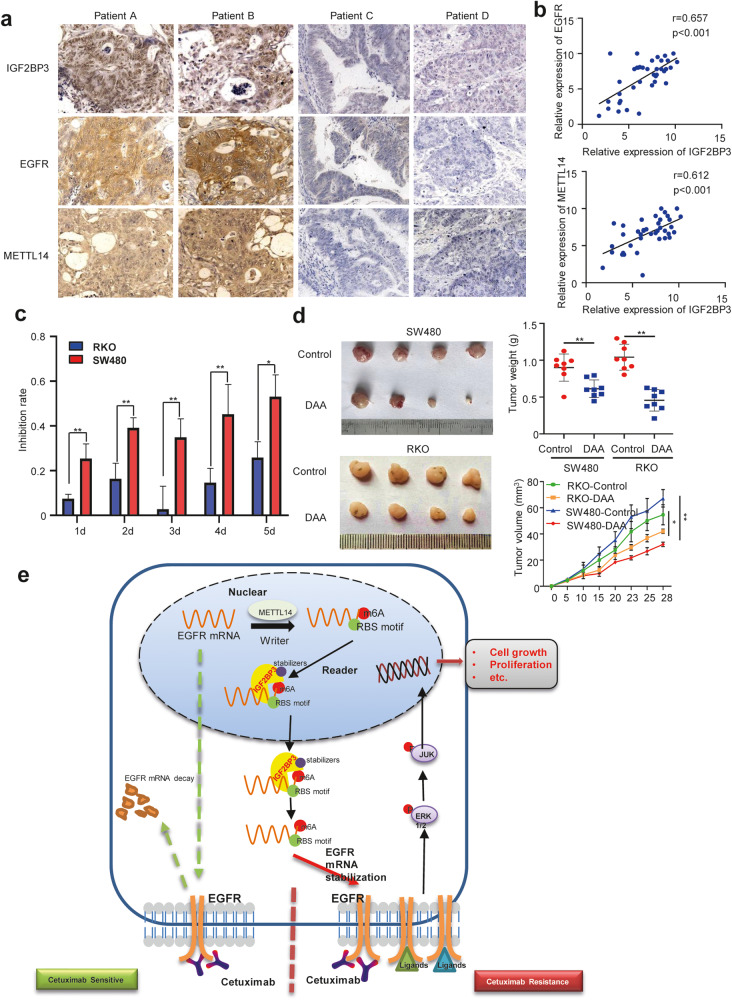
The level of IGF2BP3 is clinically relevant in CRC patients. **a** Representative IHC images of IGF2BP3, EGFR, and METTL14 in CRC tissues using IHC analysis. Scale bars: 50 μm (400X). **b** Correlation between IGF2BP3 expression and EGFR IHC scores in CRC tissues (up). Correlation between IGF2BP3 expression and METTL14 IHC scores in CRC tissues (down), *n* = 40. **c** Inhibition rate of 3-DAA (3-Deazaadenosine) (4 ug/ml) in RKO and SW480 cells. **d** Representative images of tumors (left), statistical analysis of tumor volume (right) in nude mice bearing RKO or SW480 cells with or without treatment of 3-DAA. *n* = 8. **e** Schematic diagram of the relationship among IGF2BP3, m^6^A modification, EGFR, colorectal cancer cell progression, and cetuximab resistance. Error bars represent the mean ± SD of 3 independent experiments; *p < 0.05; **p < 0.01.

We next performed the immunohistochemical staining of the CRC tissue. Samples with high IGF2BP3 expression displayed strong staining for EGFR and METTL14. In contrast, samples with low IGF2BP3 expression had low levels of EGFR and METTL14 (Fig. 6a, b).

So far, we have proved that IGF2BP3 maintains the stability of EGFR mRNA levels by combining with METTL14 in an m^6^A-dependent manner and causes drug resistance of CRC cells to cetuximab. We next hypothesized that patients with CRC having higher IGF2BP3 expression would be more sensitive to anti-tumor drug candidates targeting IGF2BP3. To test this prediction, we treated colon adenocarcinoma cell line RKO cells with a lower IGF2BP3 level with 3-deazaadenosine (DAA), an inhibitor of internal N6-methyladenosine [19]. 3-DAA treatment resulted in only 25% inhibition of cell proliferation in RKO cells. Conversely, the treatment in SW480 cells with higher expression of IGF2BP3, with the same dose of 3-DAA, resulted in an inhibition rate of 54% (Fig. 6c). These results were similar in vivo. 3-DAA was more efficient in reducing tumor weight in mice bearing SW480 cells than in those bearing RKO cells (Fig. 6d). Taken together, these data suggested that targeting m^6^A might be more effective in CRC cells with higher IGF2BP3 expression levels than in those with lower IGF2BP3 expression levels.

## Discussion

CRC accounts for approximately 10% of all annually diagnosed cancers and cancer-related deaths worldwide. Although the genetic studies of CRC have identified several alterations in crucial genes [20], it is still essential to explore additional genetic and epigenetic factors contributing to the initiation and development of CRC to identify novel therapeutic targets or biomarkers. In the present study, we confirmed the upregulation of IGF2BP3 in CRC samples and revealed that high IGF2BP3 expression was negatively correlated with clinical prognosis. We further provided evidence that the overexpression of IGF2BP3 enhanced CRC tumorigenesis and progression in vitro and in vivo and identified EGFR as an important IGF2BP3 target gene. Mechanistically, IGF2BP3 promoted EGFR expression and activated its downstream signaling by stabilizing EGFR mRNA in an m^6^A-dependent manner. We also demonstrated that high expression of IGF2BP3 increased drug resistance of CRC cells to the EGFR-targeted antibody cetuximab.

RBPs play a major role in RNA network control as regulators of the RNA life cycle from alternative splicing to nuclear export, transcript storage, stabilization, localization, and degradation [21]. The alterations in RBP expression may result in aberrant RNA translation and lead to the emergence and progression of several pathological conditions, including cancer. Among the RBPs, IGF2BP3 is of particular interest in tumorigenesis and tumor progression. IGF2BP3 was originally identified as a posttranscriptional regulator of the fetal growth factor IGF2 [22]. It is expressed during embryogenesis but absent in adult tissues. Also, it is upregulated and plays a potential oncogenic role in various tumor types. Although previous studies showed that IGF2BP3 promoted the aggressive phenotypes of CRC cells [23] and regulated cell cycle and angiogenesis in colon cancer [24], the molecular mechanisms of IGF2BP3 in CRC progression and drug resistance remain largely unclear. The present study revealed that the IGF2BP3 level was significantly upregulated in CRC tissues. Furthermore, the high IGF2BP3 expression led to worse overall survival in patients with CRC. These aforementioned findings indicated the potential value of IGF2BP3 as a biomarker for predicting CRC and as a favorable survival outcome prognostic factor, consistent with previous findings [8, 9].

Several IGF2BP3 target genes have been identified, including HK2 [25], MYC, and CDK6 [26]. Previous studies have suggested that the major mechanism for IGF2BP3 as an oncogene is to stabilize its target genes, consequently promoting the proliferation and metastasis of cancer cells. IGF2BP3 can sustain the translation of its target gene mRNA by interacting with other RNA-binding proteins [27] or noncoding RNA such as lncRNA [28] and circRNA [25], or by protecting it from miRNA-mediated decay [29]. In this study, we identified EGFR as a target gene of IGF2BP3. Further, IGF2BP3 increased EGFR expression by stabilizing its mRNA. The overexpression of IGF2BP3 facilitated the malignancy of CRC cells in vivo and in vitro. Furthermore, the inhibition of EGFR could attenuate the proliferation and progression phenotypes of IGF2BP3-overexpressing CRC cells. The immunohistochemical staining of tissues from patients with CRC further confirmed the positive correlation between IGF2BP3 and EGFR. Interestingly, previous studies have proved that EGFR signaling induces IGF2BP3 expression in MDA-MB-468 [30] and YD-10B cells [31]. Therefore, a positive feedback loop may exist between the EGFR/MEK/MAPK pathway and IGF2BP3 expression in cancer cells. Moreover, studies investigating whether other genes play essential roles in IGF2BP3-mediated cell malignancy in an m^6^A-dependent or -independent manner can enhance our understanding of the IGF2BP3-associated regulatory network.

M^6^A is the most abundant internal modification in mRNAs of eukaryotic cells [32]. It regulates almost every aspect of mRNA metabolism, from expression and pre-mRNA processing in the nucleus to translation and mRNA decay in the cytoplasm [33]. Studies have shown that the aberrancies in m^6^A are associated with various diseases including cancers [34]. M^6^A writers, erasers, and readers are proteins that can, respectively, install, remove, and recognize the m^6^A motif on mRNAs. They have been considered to be essential for tumorigenesis and tumor progression. As readers, IGF2BPs tend to identify target genes with m^6^A modifications and regulate their expression by promoting mRNA stability [3]. Similarly, the results of this study demonstrated that the overexpression of IGF2BP3 substantially lengthened the half-life of EGFR mRNA. Using m^6^A dot-blot, luciferase reporter assay, and MeRIP assay, we confirmed that, besides serving as a traditional RNA-binding site, IGF2BP3 also bound to m^6^A on EGFR mRNA to regulate its stability. A combination of previous studies and bioinformatics analysis revealed that IGF2BP3 preferentially bound to the m^6^A core motif GGAC. M^6^A is installed by a multicomponent methyltransferase complex consisting of methyltransferase like 3 (METTL3), METTL14, Wilms tumor 1 associated protein (WTAP), and so forth [33]. Our data identified METTL14 as one possible corresponding m^6^A writer. This finding was consistent with a previous study identifying METTL14 as an m^6^A writer of pleckstrin homology-like domain, family B, member 2 (PHLDB2) mRNA, which resulted in EGFR stabilization and cetuximab resistance [35]. Although METTL14 does not have catalytic activity, it plays an important role as an adapter for oncogene METTL3 to enhance the activity of methyltransferase [36]. A previous study showed that RNA binding motif protein 15 (RBM15)-mediated m6A modification of Transmembrane BAX inhibitor motif containing 6 (TMBIM6) mRNA enhanced its stability through the IGF2BP3-dependent pathway [37], suggesting that, besides METTL14, other corresponding m6A writers might exist, warranting further research.

The development of targeted therapies has provided new options for the personalized management of patients with advanced solid tumors. mAbs directed against EGFR, such as cetuximab and panitumumab, have emerged as important therapeutic agents in treating patients with metastatic CRC with all-RAS (rat sarcoma viral oncogene homolog, RAS) wild-type tumors. Nevertheless, almost all patients with tumors developed drug resistance after a few months of anti-EGFR monoclonal antibody treatment. It might be related to secondary mutations of key molecules in the EGFR pathway, especially secondary mutations of KRAS [38]. However, some patients without secondary mutations in KRAS also developed resistance to anti-EGFR monoclonal antibody therapy; however, the specific molecular mechanism is not yet clear. Our study was novel in demonstrating that IGF2BP3 was associated with drug resistance to the EGFR-targeted antibody cetuximab in CRC cells. IGF2BP3 can stabilize EGFR mRNA through m^6^A modification when KRAS is wild type, thereby continuously activating the downstream signaling pathway of EGFR, promoting tumorigenesis and evolution of CRC, and then affecting the sensitivity of patients with CRC to anti-EGFR monoclonal drug therapy. This finding is consistent with previous studies identifying drugs and miRNAs that downregulate EGFR expression in cancer cells and potentiate the effects of cetuximab [39, 40]. Our studies imply a critical role of IGF2BP3 in predicting the prognosis of cetuximab treatment. Moreover, IGF2BP3 might holds the potential as a feasible target for overcoming cetuximab drug resistance. Recent research has identified rigosertib as a potent small molecule that can suppress tumorigenesis by downregulating IGF2BP3 [41]. Non-coding RNAs such as miR-486 [42] and gga-miR-449b-5p [43] are also attractive alternative approaches to target IGF2BP3. More detailed preclinical studies are required before these drugs can be considered a concrete possibility.

Previous studies on the role of IGF2BP3 indicated that, as a binding protein of IGF2, one of the main functions of IGF2BP3 is the enhancement of IGF2 translation [16]; it then exerts its oncogenic functions through the translational activation of the IGF2 mRNA [44]. On the contrary, some studies have shown that the overexpression of IGF2 is the major tumorigenic driver in a subset of CRCs [45] and correlates with reduced sensitivity to cetuximab in patients with CRC [46]. However, recent studies have shown that the IGF2BP3 protein can bind to mRNAs such as IGF2 and C-myc not only in an IGF2-dependent manner but also in an IGF2-independent manner, such as an m6A reading protein, thereby stabilizing target genes and promoting tumor cell proliferation, invasion, and chemotherapy resistance. In our study, we also added a corresponding rescue experiment to confirm that IGF2BP3 directly targeted EGFR in an m6A-dependent manner to promote CRC progression and drug resistance, which weakened the influence of other factors on the experimental results.

In short, IGF2BP3 plays an oncogenic role in stabilizing EGFR mRNA in an m^6^A-dependent manner and further regulates cancer cell proliferation and drug resistance to cetuximab in CRC cells (Fig. 6e). Besides IGF2BP3 biological and epigenetic importance, our work may be relevant to the clinical management of patients with CRC, as IGF2BP3 may be a promising biomarker to guide early diagnosis and therapy of CRC. We further found that 3-DAA, a chemical inhibitor of internal N6-methyladenosine, is more effective in inhibiting cell proliferation in CRC cells with higher IGF2BP3 expression than in cells with lower IGF2BP3 expression. 3-DAA also inhibits other signaling pathways [47, 48], and hence a more specific inhibitor of IGF2BP3 needs to be developed for patients with CRC. Therefore, the combined treatment with anti-EGFR drugs, such as cetuximab, and with specific inhibitors for IGF2BP3 or m^6^A, can be a potential therapeutic strategy to investigate in a clinical setting for overcoming intrinsic or acquired resistance to EGFR inhibitors in patients with CRC.

## Materials and Methods

### Clinical samples

Paraffin samples of 67 CRC tissues with complete clinical data were obtained from Nanfang Hospital, Southern Medical University. A total of 47 CRC tissues and adjacent normal mucosa tissues were also collected from CRC patients undergoing standard resection, without chemotherapy or radiotherapy immunotherapy, at Nanfang Hospital. All specimens were histopathologically confirmed by at least two pathologists and obtained with informed consent. This study was approved by Southern Medical University Institutional Board (Guangzhou, China).

### Cell culture and treatment

The human colorectal cancer cell lines SW480, HCT116, RKO, and CaCO2 were obtained from the American Type Culture Collection (ATCC). SW620 and HT29 cells were cultured in DMEM medium (Gibco) with 10% FBS (Gibco) at 37 °C with 5% CO2. SW480, Colo205, Ls174t, HCT116, LoVo, HCT15, RKO, and CaCO2 cells were cultured in RPMI 1640 medium (Gibco) with 10% FBS (Gibco) at 37 °C with 5% CO2. All cell lines were routinely authenticated for purity and being infection-free.

### Quantitative real-time PCR (qRT-PCR)

Total RNA was isolated from CRC tissues and cell lines using Trizol reagent (Invitrogen). 1 μg total RNA was reverse transcribed into cDNA by PrimeScript RT reagent Kit (Takara). The acquired cDNAs were used as templates for quantitative real-time PCR analysis using ChamQTM Universal SYBR Q-PCR Master Mix (Vazyme). The relative RNA expression levels were calculated using the 2^-ΔΔCt^ method, with the levels normalized to GAPDH mRNA. Sequences of the real-time PCR primers were: IGF2BP3 (Forward, 5′-TATATCGGAAACCTCAGCGAGA-3′; Reverse, 5′- GGACCGAGTGCTCAACTTCT-3′); METTL14 (Forward, 5′- AGTGCCGACAGCATTGGTG-3′; Reverse, 5′- GGAGCAGAGGTATCATAGGAAGC-3′); GADPH (Forward, 5′-ACAGTCAGCCGCATCTTCTT-3′; Reverse, 5′-GACAAGCTTCCCGTTCTCAG-3′);

### Western blotting

Treated CRC cells were extracted by RIPA lysis buffer and quantified by BCA Protein Assay Reagent Kit (Thermo Fisher Scientific). 20–50 μg of protein was separated by SDS-polyacrylamide gels and then transferred to PVDF membranes (Millipore). The membranes were blocked with 5% nonfat milk for 1–2 h and incubated with primary antibody at 4°C overnight. Anti-α-tubulin monoclonal antibody (T6199; Sigma) was used as a loading control. The primary antibodies were IGF2BP3 (Abcam, ab208869, 1:400), EGFR (Abcam, ab52894, 1:1000), ERK (Cell Signaling Technology, #4695 S, 1:200), p-ERK (Cell Signaling Technology, #4370 S, 1:200), JNK (Cell Signaling Technology, #9251 S, 1:200), p-JNK (Cell Signaling Technology, #9255 S, 1:200), METTL14 (Solarbio, K107984P, 1:4000). Secondary antibodies (1:8000) were labeled with HRP. The ECL detection system (KeyGEN) was used for visualization.

### Histology and immunohistochemistry (IHC)

Tissue samples were fixed with 4% paraformaldehyde. After incubation, the samples were washed and dehydrated in graded ethanol. After appropriate permeation in xylene, the fixed tissues were embedded in paraffin and followed by cutting 2.5 μm paraffin sections. Then they were de-paraffinized twice with xylene and rehydrated in descending concentration of ethanol. Standard hematoxylin-eosin (HE) staining of paraffin-embedded tissues was used for histological examination.

The sections along the HE stained slides were further processed for IHC. The protein expression levels of IGF2BP3, EGFR, and METTL14 were determined by IHC analysis. Briefly, the slides were incubated with primary antibodies: IGF2BP3 (Altas Antibodies, HPA002037, 1:200), EFGR (Abcam, ab52894, 1:1000), METTL14 (Solarbio, K107984P, 1:50), at 4 °C overnight. The subsequent steps were performed using the SP-9000 SPlink Detection Kit (ZSGB-BIO) according to the manufacturer’s instructions. The final stainings were scored according to immunoreactivescore (IRS) analysis: staining intensity (SI), 0 (no staining), 1 (weak), 2 (moderate), or 3 (strong); percentage of positive cells (PP), 0 (negative staining), 1 (1%–10% positive staining), 2 (10–25% positive staining), 3 (25–50% positive staining), and 4 (50–75% positive staining). SI and PP were multiplied to give a final score. The tissues with a final score ≤3 were classified as ‘Low Expression’ and the tissues with a final score >3 were classified as ‘High Expression’.

### Establishment of Stable IGF2BP3 and EGFR knockdown and overexpression cells

The IGF2BP3 and EGFR constructs were generated by subcloning PCR amplified full-length human IGF2BP3 and EGFR cDNA into pEZ-Lv105. Stable knockdown of target genes was achieved by lenti-viral based short-hairpin RNA delivery. Target specific shRNAs were cloned into the lenti-viral vector pLKO.1. Viral particles were packaged in 293FT and used to infect SW480 and HCT116 cell lines. Infected cells were selected by puromycin and expanded to form a stable sub-line. Knockdown efficiency was confirmed at both mRNA and protein levels. A non-target control shRNA purchased from Sigma-Aldrich was used as a negative control. The target sequence of IGF2BP3 was shRNA1 (Forward, 5′- CCGGCGGTGAATGAACTTCAGAATTCTCGAGAATTCTGAAGTTCATTCACCGTTTTTG - 3′; Reverse, 5′- AATTCAAAAACGGTGAATGAACTTCAGAATTCTCGAGAATTCTGAAGTTCATTCACCG-3′); shRNA2 (Forward, 5′- CCGGGCAGGAATTGACGCTGTATAACTCGAGTTATACAGCGTCAATTCCTGCTTTTTG-3′; Reverse, 5′- AATTCAAAAAGCAGGAATTGACGCTGTATAACTCGAGTTATACAGCGTCAATTCCTGCTTTTTG -3′);

### Establishment of IGF2BP3 knockout cells

IGF2BP3 knockout in SW480 cells was generated using CRISPR/Cas9 technology by lentiviral transduction and puromycin selection. Cas9 and single guide RNAs (sgRNAs) lentivirus were bought from Genechem. SW480 cells were first engineered to express Cas9. sgRNAs targeting IGF2BP3 were cloned into the lentiCRISPR vector. SW480 cells were transduced with lentiCRISPR-IGF2BP3 and selected with puromycin. The knockout cell clones were isolated by limited serial dilution. A monoclonal cell line was generated, and the IGF2BP3 knockout effect was confirmed using Western blot and sequencing analysis. The sgRNA sequence targeting IGFBP3 was listed as follows: sg1 (Forward, 5′-CTAAACTTCCATGTTGGCTATTATTG-3′; Reverse, 5′-CAATATTTGGTTTCTTATCCCAAAG-3′); sg2 (Forward, 5′- GAAATGGCCGCCCAGCAAAACC-3′; Reverse, 5′- CACATTCCCAAGTACTGTACAAG-3′). A non-target control shRNA was used as a negative control.

### In vitro cell proliferation, migration, and invasion assays

A colony-forming assay was used to determine the proliferation of cells. Cells were seeded in 6-well culture plates at a concentration of 200 cells per well. The cells were cultured for 14 days and then washed two times with PBS, fixed in 4% (vol/vol) paraformaldehyde, and stained with 0.1% (wt/vol) crystal violet solution. The colonies were counted under the microscope (1 colony for more than 50 cells). For the MTT assay, 1000 cells were seeded and transfected in a 96-well plate. After 24 h, 5 mg/ml MTT solution was added to each well and the cells were incubated with MTT for 4 h in standard conditions. Then, the supernatant was removed. In all wells, 10 μl DMSO for MTT crystals dilution were added. The absorbance was measured at 570 nm using an automated microplate reader. For soft agar assay, each well of a six-well culture dish was coated with 2 ml bottom agar mixture (DMEM or 1640, 1.32% (w/v) agar). After the bottom layer had solidified, 2 ml top agar-medium mixture (DMEM or 1640, 0.66% (w/v) agar) containing 1 × 10^5^ cells was added, and the dishes were incubated at 37 °C for 2 weeks. The plates were stained with Crystal Violet, then the number of colonies was counted by a dissecting microscope. Experiments were repeated three times.

### Mouse experiments

In vivo experiments were all performed on 4–6-week-old female BABL/cAnN-nude mice bought from Southern Medical University Animal Center. For the subcutaneous implantation model, 2 × 10^6^ cells were first suspended in 200 ul serum-free medium and then were injected subcutaneously in the right flank. After one week, the tumor volumes were measured every day. Mice were sacrificed at week 4 to harvest the tumor bulks. For in vivo cetuximab treatment, one week after subcutaneous implantation, mice were randomized into control (0.9% NaCl), low dose cetuximab (1 mg/kg), and high dose cetuximab (2 mg/kg) groups, intraperitoneal (i.p.) injection, every 3 days. Mice were sacrificed at week 14 to harvest the tumor bulks. Tumor volume was calculated by the formula V = ab^2^/2, where a and b are the tumor’s length and width, respectively.

### Luciferase reporter assay

The fragments of EGFR-3’UTR containing the wild-type m^6^A motifs as well as mutant m^6^A motifs (m^6^A was replaced by T) were synthesized at HanYi Biology Technologies. The wild-type and mutant EGFR-3’UTR fragments were inserted into the upstream of pmirGLO-basic firefly luciferase vector. CRC cells were seeded in 24-well plates and transfected with 5 ug wild-type or mutant EGFR-3’UTR. The relative luciferase activity was detected using the Dual-Luciferase Reporter Assay System (Promega) 48 h later. Firefly luciferase activity and Renilla luciferase activity were measured using Synergy NE02 (BioTek). The results were shown in the form of relative firefly luciferase activity normalized to Renilla luciferase activity. All the experiments were repeated three times, and three replicates were conducted for each group.

### mRNA stability analysis

Cells with or without IG2BP3 overexpressed and knockout were directly treated with Actinomycin D for 0 h, 3 h, and 6 h at a final concentration of 5 μg/mLand harvested at the indicated time points. Total RNA was extracted, and real-time PCR was conducted to quantify the relative level of EGFR mRNA. The degradation rate and half-life of EGFR mRNA were estimated according to the published paper. Briefly, the degradation rate of mRNA (K_decay_) was calculated by the following equation:

$${\rm{ln}}({\rm{C}}/{\rm{C}}0)=-{K}{_{{{dacay}}}{t}}$$

*t* is the transcription inhibition time, and C is the mRNA level at the time *t*. C_0_ is the level of mRNA at 0 h in the equation, which means the mRNA level before decay starts. Thus, the mRNA half-time (t_1/2_) can be calculated by the equation

$${\rm{l}}{\rm{n}}(1/2)=-{K}{_{{decay}}}t{_{1/2}}$$

### m^6^A dot blot

Polyadenylated mRNA was purified by GenEluteTM mRNA Miniprep Kit (Sigma) from previously isolated total RNA. The poly(A) + RNA samples were loaded to Hybond-N+ membrane (GE Healthcare) and UV crosses with the nylon membrane. The membrane was then blocked with 5% nonfat milk for 1 h and incubated with m^6^A antibody (Abcam, 190886) at 4 °C, overnight. After incubating with horseradish peroxidase-conjugated anti-mouse IgG, the membrane was visualized with the ECL detection system. The relative signal density of each dot was quantified by Image J software. The results of the m^6^A level were shown in the form of relative m^6^A dot blot density normalized to methylene blue staining density.

### MeRIP and MeRIP-qPCR

Total RNA was extracted by Trizol reagent (Takara), and mRNA was purified using GenEluteTM mRNA Miniprep Kit (Sigma). RNA fragmentation reagents (NEB) were used to randomly fragment RNA. The specific anti-m^6^A antibody (NEB) was applied for m^6^A immunoprecipitation. Anti-m^6^A antibody was pre-bound to Protein G magnetic beads in reaction buffer for 30 min. The fragmented mRNA was incubated with m^6^A-antibody-bound protein G magnetic beads at 4 °C for 1 h and washed with low salt reaction buffer and high salt reaction buffer. M^6^A-antibody-bound RNA was extracted from the Dynabeads using Buffer RLT (Qiagen) and further incubated with Dynabeads MyOne Silane (Life Technologies). The RNA and Dynabeads mixture was precipitated with 100% ethanol washed with 70% ethanol and then re-suspended with nuclease-free water. The supernatant was carefully collected after the beads were pulled to the side of the tube by a magnetic field. Real-time PCR was carried following m^6^A-IP to quantify the changes to m^6^A methylation of EGFR. Sequences of the real-time PCR primers of EGFR were: forward, 5′- GATGGGCAGGTCAGGAGA-3′; reverse, 5′- CCAGGCTATCAATCAGGAAG-3′.

### Electrophoretic Mobility Shift Assay (EMSA)

The validation of mRNA-protein interactions was performed using the Chemiluminescent EMSA Kit (Beyotime) according to the manufacturer’s protocol. For the binding assays, the following oligonucleotides were designed and used: specifically bound biotin-labeled probes, specifically bound unlabeled probes, and independent unlabeled mutation probes. All oligonucleotides were designed based on the results of the previous double luciferase experiment and purchased from Generay.

### 3-DAA (3-Deazaadenosine) inhibit experiment

The 3-DAA inhibit experiments were performed as described previously [49]. Cells were seeded in 96-well culture plates. 4ug/ml 3-DAA (APExBIO, B6121) were added. MTT assay was then performed to calculate the inhibition rate.

### Statistical analysis

Statistical analyses were carried out using SPSS 20.0. Data are presented as means ± SD from three independent experiments. For continuous variables, data were analyzed by two-tailed unpaired Student’s t-test between two groups and one-way ANOVA for multiple comparisons. For categorical variables, Chi-square test was used. The overall survival rate curves based on the Kaplan-Meier method were plotted using the log-rank test. The correlation between IGF2BP3 expression and EGFR and METTL14 expression was determined by Spearman correlation analysis. P values less than 0.05 were considered statistically significant.

### Reporting summary

Further information on research design is available in the Nature Research Reporting Summary linked to this article.

## Supplementary information

Supplementary Figure S1

Supplementary Figure S2

Supplementary Figure S3

Supplementary Figure S4

Supplementary Figure S5

Supplementary Table S1

Supplementary Figure Legend

Western Blot

Reporting Summary

## Data Availability

The high-throughput RNA sequencing data were deposited by the National Natural Science Foundation of China (Grant No. U1201226). All other data supporting the findings of this study are available within the article and its Supplementary Information files and from the corresponding author upon reasonable request.
