# Supplementary figures and images for: IGF2BP3 promotes the progression of colorectal cancer and mediates cetuximab resistance by stabilizing EGFR mRNA in an m6A-dependent manner
Source: Cell Death Dis. 2023 Sep 1;14(9):581. doi: 10.1038/s41419-023-06099-y (PMC10474290; doi:10.1038/s41419-023-06099-y)

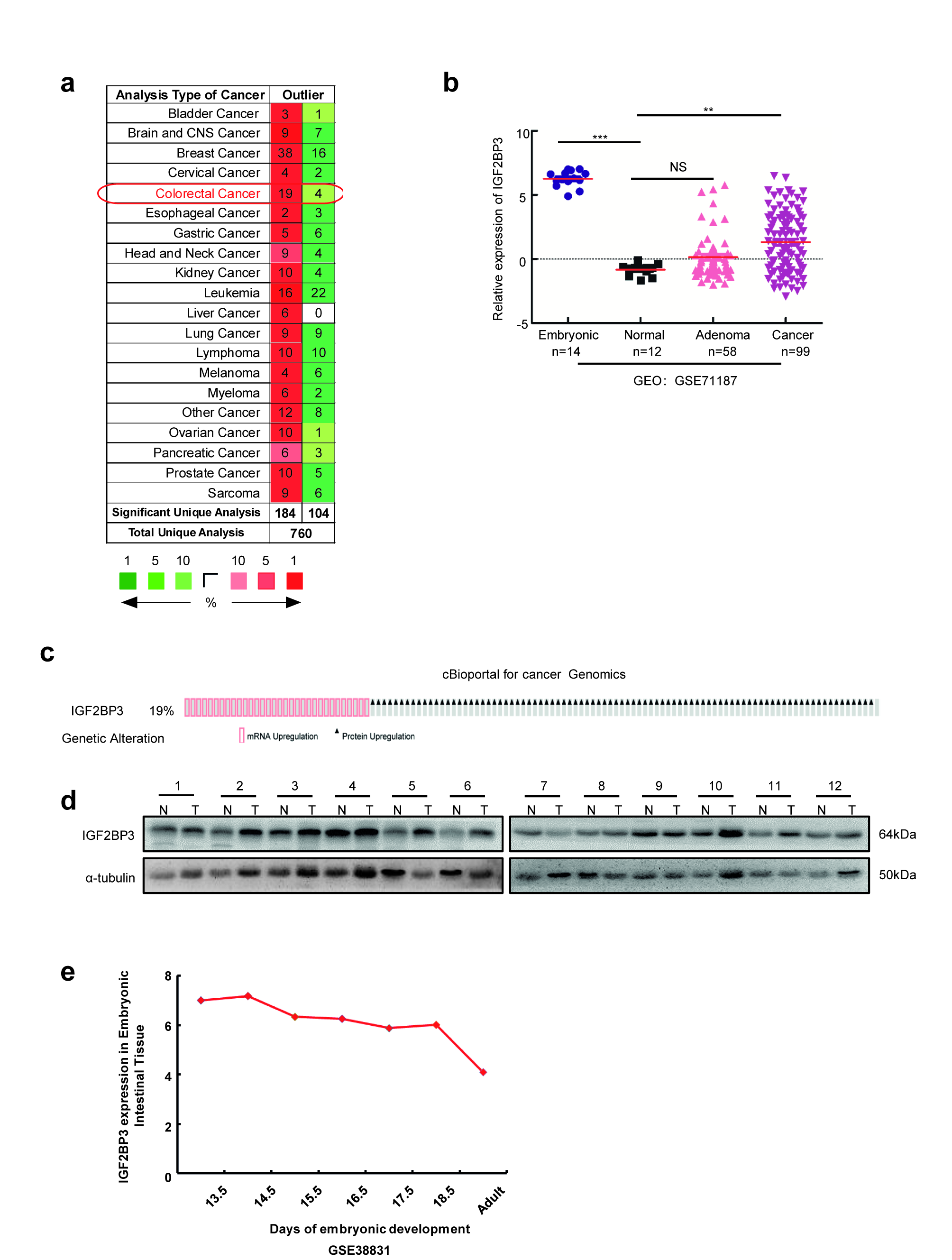

Supplement: Supplementary file 1 — Supplementary Figure S1 [file 41419_2023_6099_MOESM1_ESM.tif]

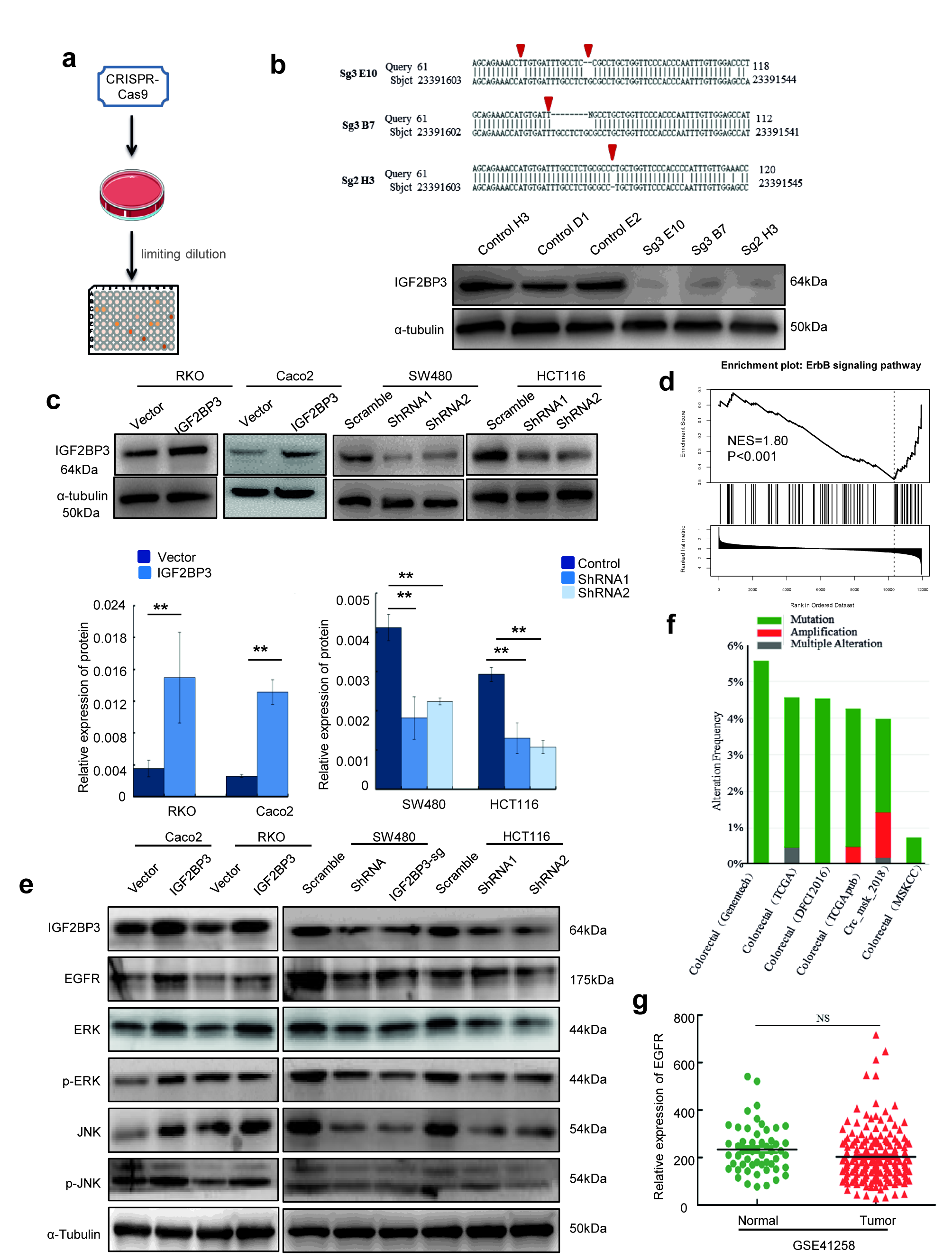

Supplement: Supplementary file 2 — Supplementary Figure S2 [file 41419_2023_6099_MOESM2_ESM.tif]

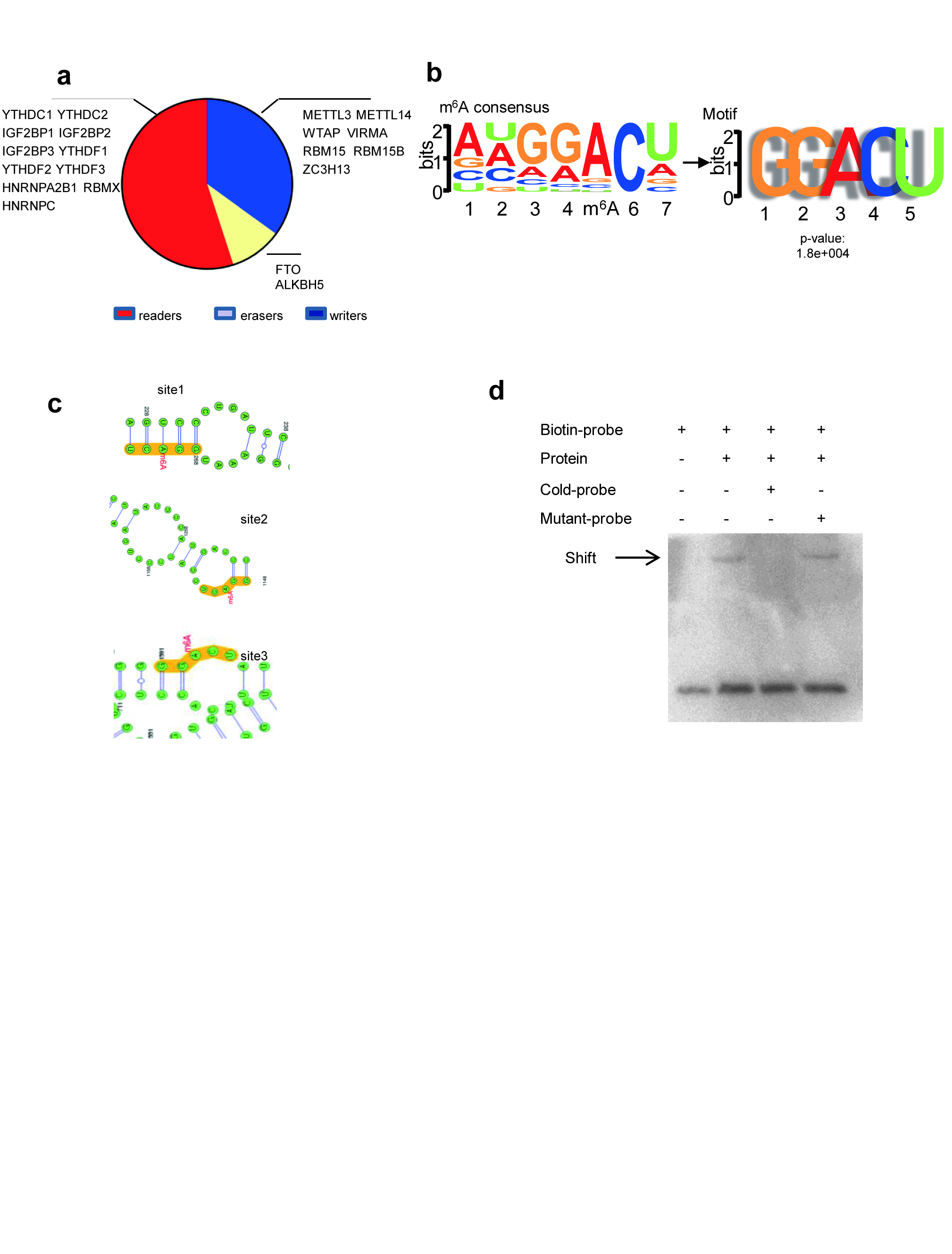

Supplement: Supplementary file 3 — Supplementary Figure S3 [file 41419_2023_6099_MOESM3_ESM.tif]

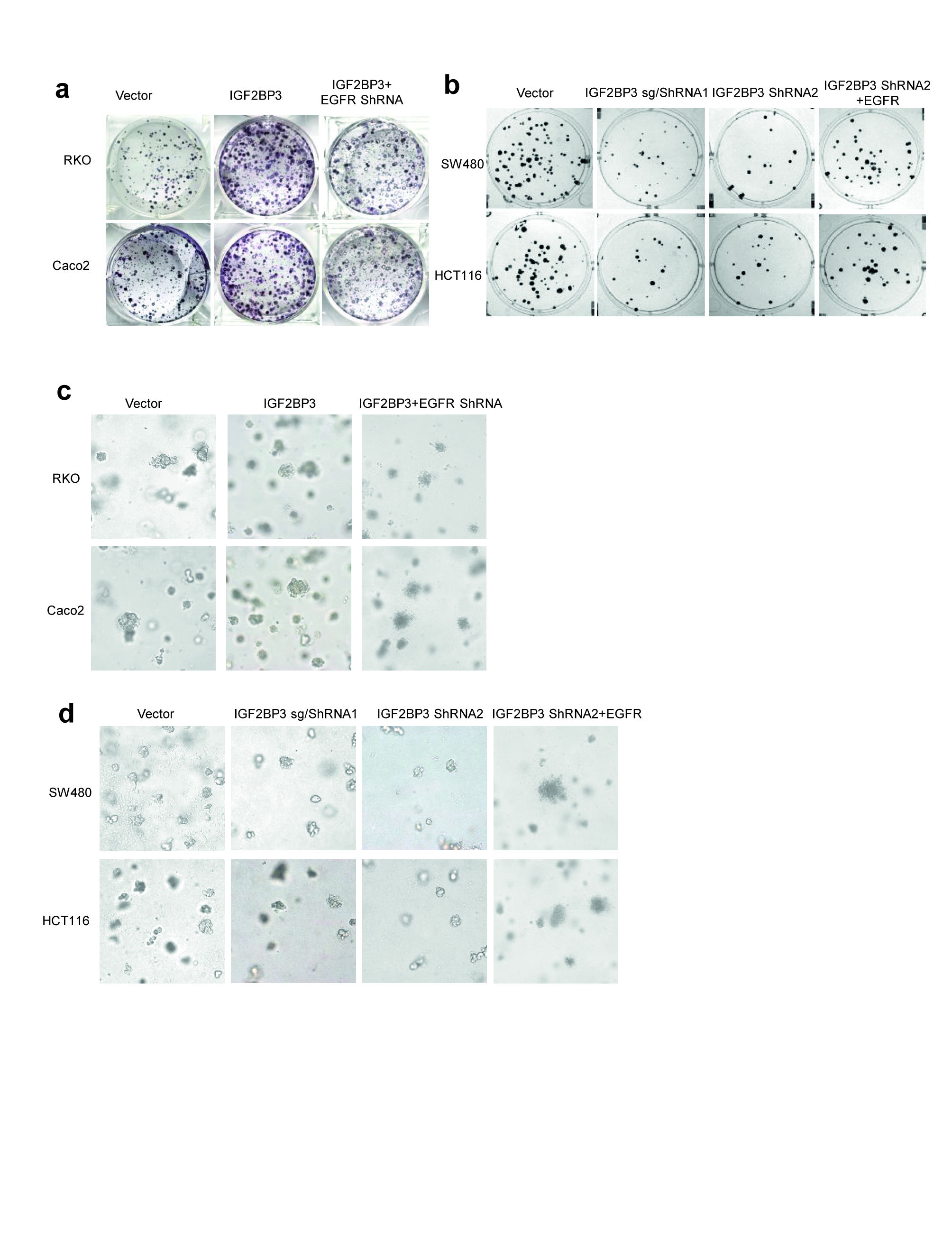

Supplement: Supplementary file 4 — Supplementary Figure S4 [file 41419_2023_6099_MOESM4_ESM.tif]

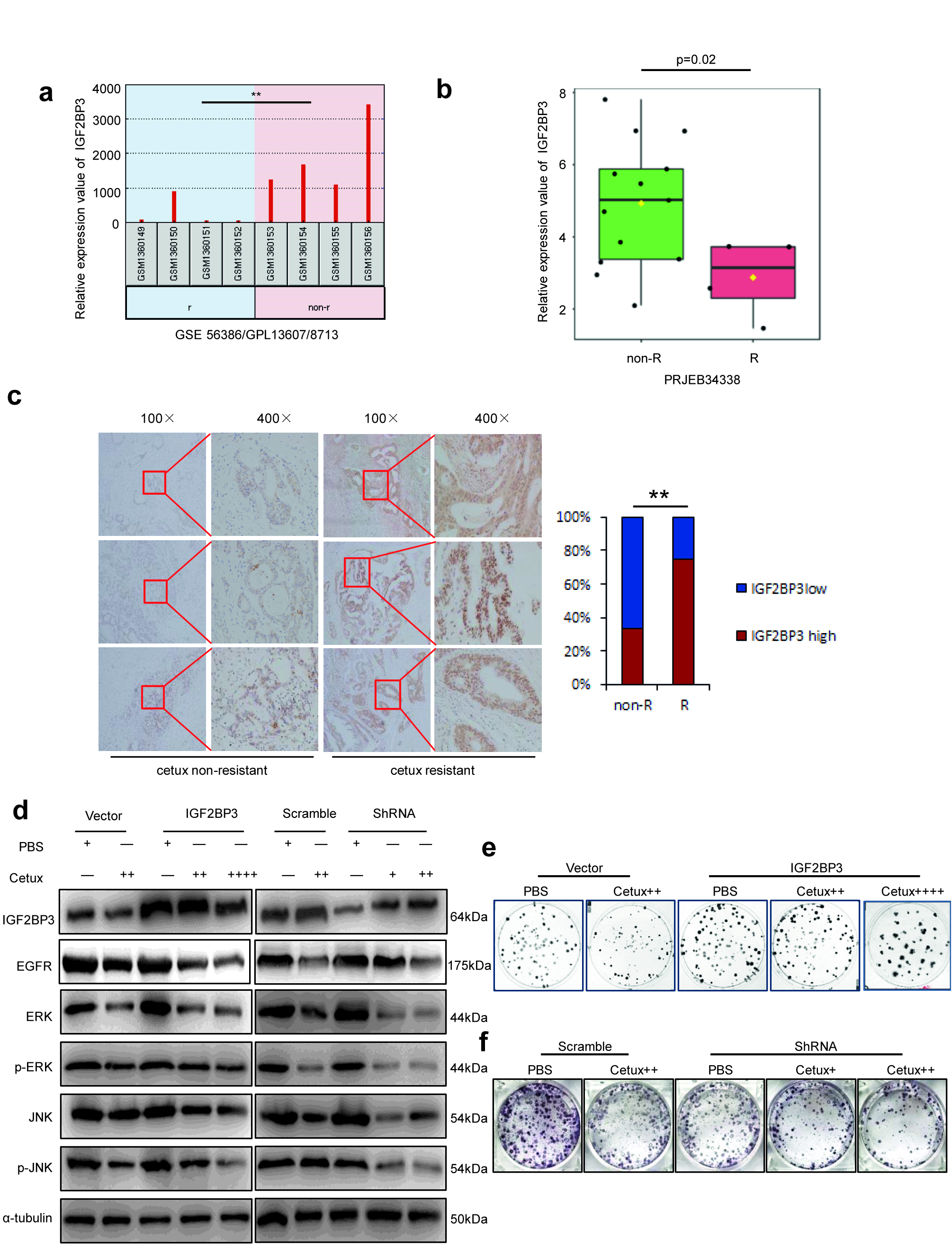

Supplement: Supplementary file 5 — Supplementary Figure S5 [file 41419_2023_6099_MOESM5_ESM.tif]

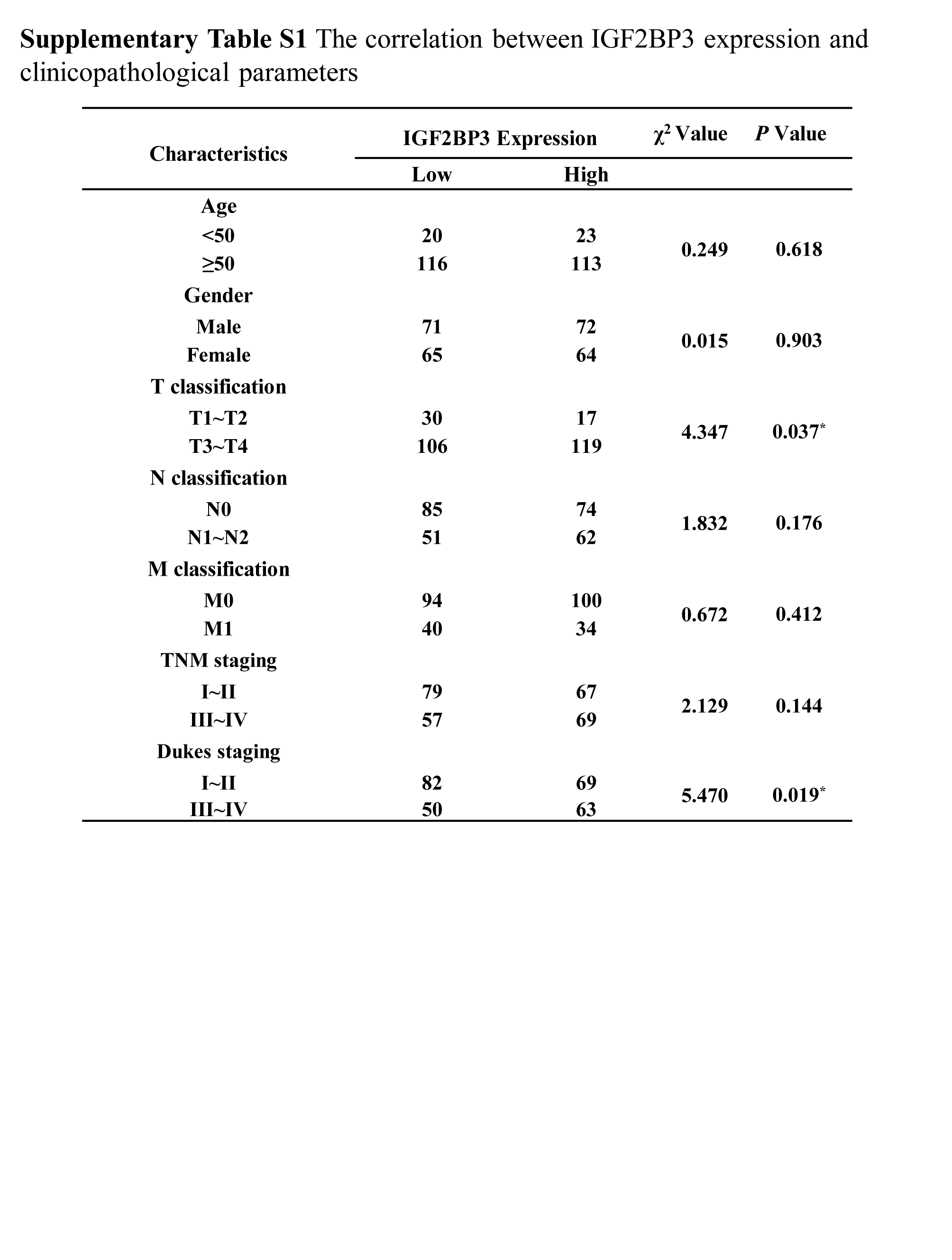

Supplement: Supplementary file 6 — Supplementary Table S1 [file 41419_2023_6099_MOESM6_ESM.tif]
