# Supplementary material for: IGF2BP3 promotes the progression of colorectal cancer and mediates cetuximab resistance by stabilizing EGFR mRNA in an m6A-dependent manner
Source: Cell Death Dis. 2023 Sep 1;14(9):581. doi: 10.1038/s41419-023-06099-y (PMC10474290; doi:10.1038/s41419-023-06099-y)
Supplement: Supplementary file 7 — Supplementary Figure Legend [file 41419_2023_6099_MOESM7_ESM.docx]

**Fig. S1 IGF2BP3 is up-regulated in colorectal cancer. a** Expression of IGF2BP3 in Oncomine Database in Colorectal Cancer and Other Tumor Database. **b** Expression of IGF2BP3 in embryonic, normal, adenoma, and cancer tissue from GSE71187 dataset. **c** Expression of IGF2BP3 in cBioPortal for Cancer Genomics databases. **d** Western blot analysis of IGF2BP3 in 12 cases of fresh CRC tissues. **e** Expression of IGF2BP3 in mouse embryonic intestine tissue in different days of embryonic development from GSE38831 dataset. N, normal. T, tumor. ns, no significance; ** p < 0.01; *** p < 0.001;

**Fig. S2 IGF2BP3 regulates the stability of EGFR mRNA. a** Schematic depicting the establishment of IGF2BP3 stably knockout monoclonal SW480 cell lines. **b** Target gene sequence identification (Up) and protein identification (Down) of IGF2BP3 stably knockout monoclonal cell lines. **c** The overexpression and knockdown efficiency of IGF2BP3 was confirmed by Western blot. **d** GSEA analysis of the ErBb signaling pathway in IGF2BP3 knockout and control SW480 cells. **e** Western blot analysis of the expression of EGFR signaling pathway in IGF2BP3 overexpression and knockdown/knockout cell lines. **f** The alteration frequency of EGFR in colorectal cancer by cBioprotal. **g** Relative expression of EGFR mRNA in GSE41258. ** p < 0.01;

**Fig. S3 IGF2BP3 stabilizes EGFR mRNA in an m6A-dependent manner. a** The proportion of readers, writers, and erasers of m^6^A regulators. **b** Previous study (left) and HOMER motif analysis (right) both revealed the top consensus m^6^A motif ‘GGAC’. **c** The top three most likely m^6^A binding sites of IGF2BP3 protein and the 3'UTRs of EGFR mRNA predicted by bioinformatics. **d** Electrophoretic mobility shift analysis of interactions between EGFR mRNA and IGF2BP3.

**Fig. S4 IGF2BP3 induces tumor cell proliferation and tumorigenesis depending on the expression of EGFR in CRC. a-d** The ability of proliferation of CRC cells were evaluated by the colony formation assay (**a**, **b**), soft agar assay (**c**, **d**) in the indicated cells with different treatments.

**Fig. S5 IGF2BP3 affects the sensitivity of colorectal cancer cells to cetuximab. a** Expression of IGF2BP3 in response (r) (4 cases) and non-response (non-r) (4 cases) to cetuximab from GSE56386 dataset using GEO2R. **b** Expression of IGF2BP3 in response (r) (13 cases) and non-response (non-r) (4 cases) to cetuximab from PRJEB34338 dataset using NetworkAnalyst. **c** Representative IHC images of IGF2BP3 in cetuximab non-resistant and cetuximab resistant CRC tissues. **d** Western blot analysis of the expression of IGF2BP3, EGFR and its downstream effector molecules in indicated groups of Caco2 cells. **e, f** Colony formation assay of Caco2 cells validated the effect of different cetuximab treatments on the proliferation rate in indicated groups.
