# Supplementary material for: IGF2BP3 promotes the progression of colorectal cancer and mediates cetuximab resistance by stabilizing EGFR mRNA in an m6A-dependent manner
Source: Cell Death Dis. 2023 Sep 1;14(9):581. doi: 10.1038/s41419-023-06099-y (PMC10474290; doi:10.1038/s41419-023-06099-y)

Figure 3a

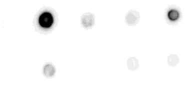

Figure 3f

IGF2BP3

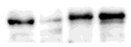

METTL14

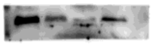

β-actin

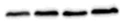

Figure S1d

IGF2BP3 1-6

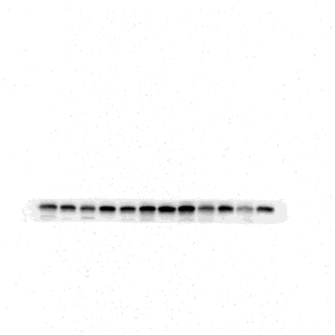

IGF2BP3 7-12

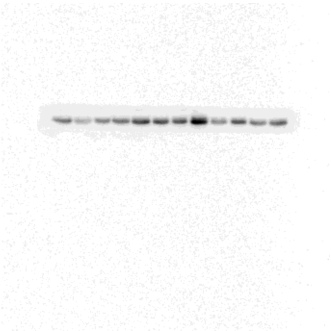

α-tublin 1-6

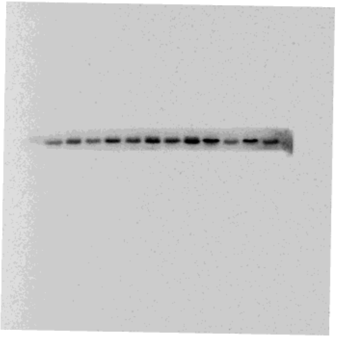

α-tublin 7-12

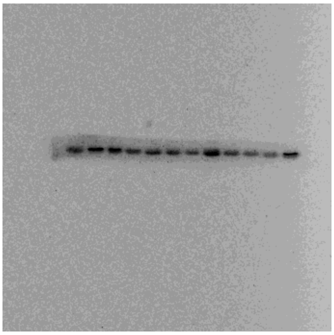

Figure S2b

IGF2BP3

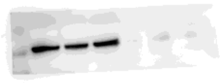

α-tublin

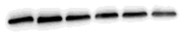

Figure S2c

SW480 IGF2BP3

SW480 α-tublin

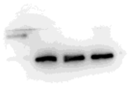

Figure S2c

Caco2

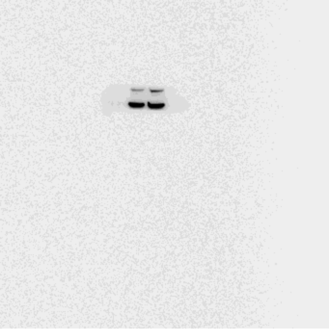

HCT116 IGF2BP3

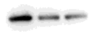

HCT116 α-tublin

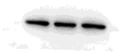

RKO IGF2BP3

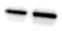

RKO α-tublin

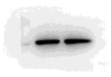

Figure S2d

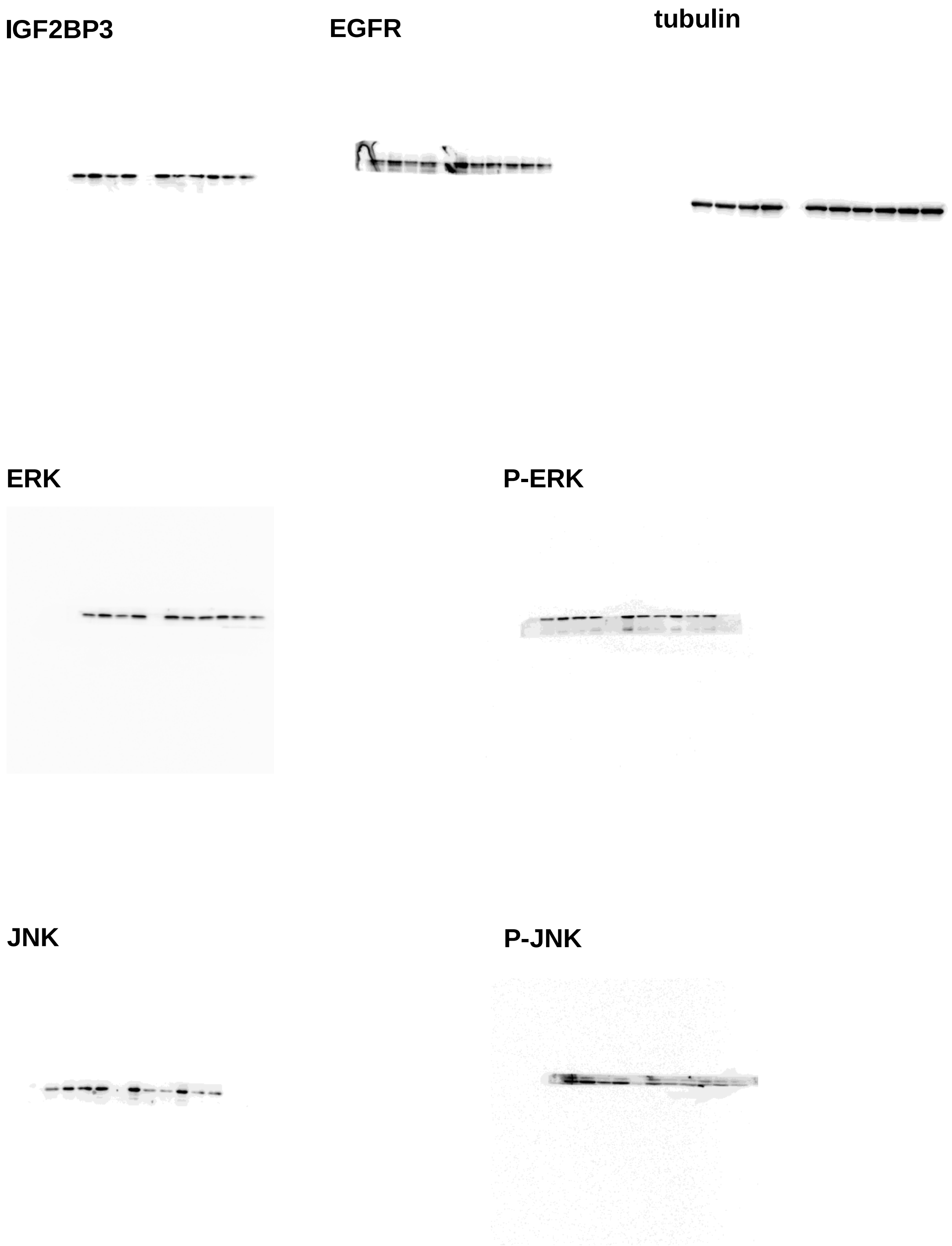

Figure S3d

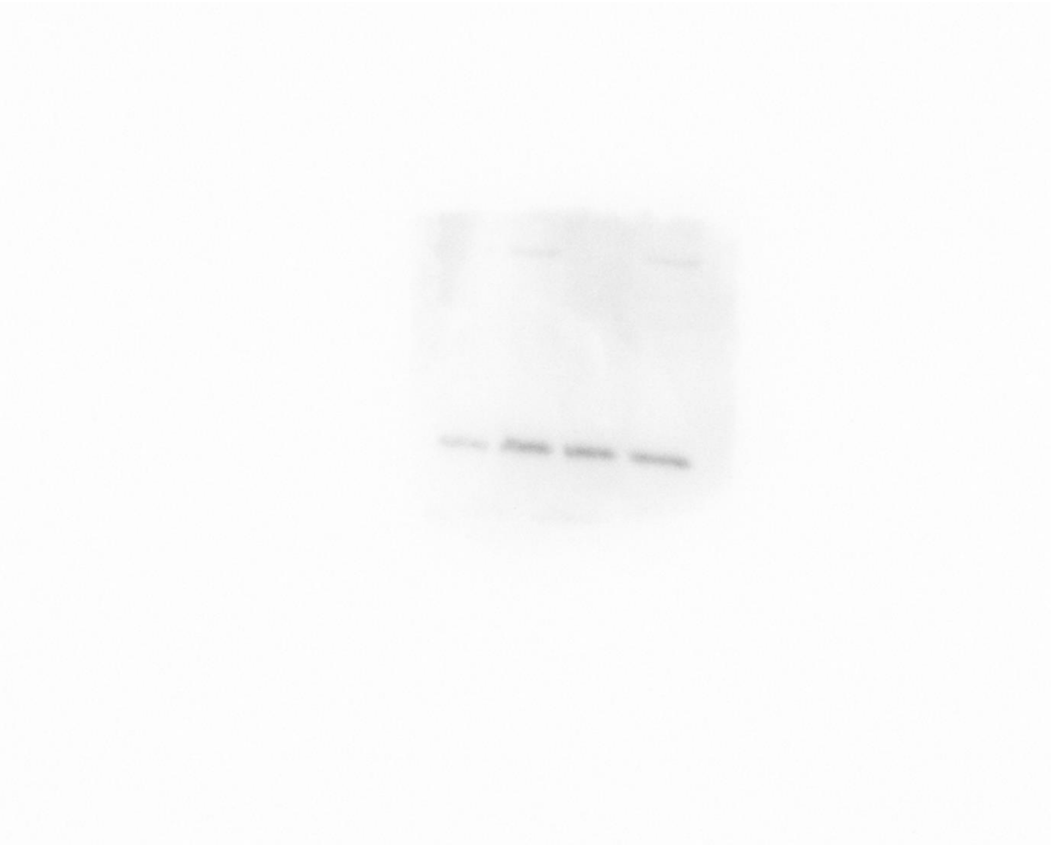

Figure S5d

IGF2BP3

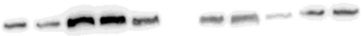

EGFR

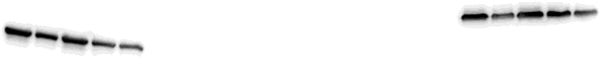

ERK

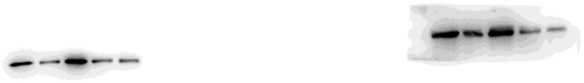

JNK

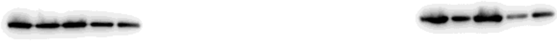

Figure S5c

P-ERK

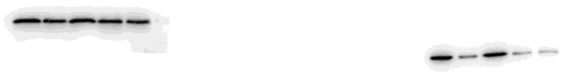

P-JNK

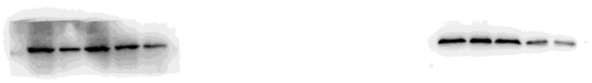

tubulin

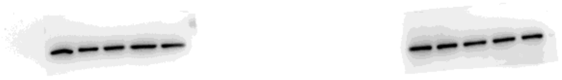

Supplement: Supplementary file 8 — Western Blot [file 41419_2023_6099_MOESM8_ESM.pdf]
